# Mortality attributable to tobacco among men in Sweden and other European countries: an analysis of data in a WHO report

**DOI:** 10.1186/1617-9625-12-14

**Published:** 2014-09-01

**Authors:** Lars Ramström, Tom Wikmans

**Affiliations:** 1 Institute for Tobacco Studies, Kanalvägen 17, Täby, 18338, Sweden; 2 Liqusa Research, Åtvidaberg, Sweden

**Keywords:** Mortality, Snus, Smoking, Sweden, Europe

## Abstract

**Background:**

It is well known that Swedish men have lower tobacco-related mortality than men in other European countries, but there are questions that need further investigation to what extent this is related to the specific patterns of tobacco use in Sweden, where use of snus, the Swedish low-nitrosamine oral tobacco, dominates over smoking in men but not in women. The recent *WHO Global Report: Mortality Attributable to Tobacco* provides a unique set of estimates of the health burden of tobacco in all countries of the world in the year 2004, and these data can help elucidating the above-mentioned questions.

**Methods:**

For Sweden and all other European Union Member States mortality data for a number of tobacco-related causes of death were extracted from the WHO Report. The size of the mortality advantage for selected causes of death in different age groups of Swedish men compared to men of the same age in Europe as a whole was calculated in terms of ratios of death rates attributable to tobacco. Differences between age groups with respect to tobacco-related mortality were analyzed with respect to differences in terms of development and status of smoking and snus use. The analyses also paid attention to differences between countries regarding tobacco control regulations.

**Findings:**

Among men in the European Union Member States the lowest level of mortality attributable to tobacco was consistently found in Sweden, while Swedish women showed levels similar to European average. A strong co-variation was found between the mortality advantage and the degree of dominance of snus use in the different age groups of Swedish men. Among Swedish women there are no age groups with dominant use of snus, and similar observations were therefore not possible for women.

**Conclusion:**

The above findings support the assumption that the widespread use of snus instead of cigarettes among Swedish men may be a major part of the explanation behind their position with Europe’s lowest mortality attributable to tobacco.

## Background

A large study of tobacco use in European Union Member States around 2002 found that the proportion of daily users of tobacco among adult men in Sweden was about the same as the average among men in the EU as a whole [1]. But, Swedish men are unique in their choice of tobacco products. Most of them use use snus, the Swedish kind of low-nitrosamine moist snuff, while cigarette smokers are a minority. This means that the prevalence of daily smoking in men is lower in Sweden than in other EU countries.

The *WHO Global Report: Mortality Attributable to Tobacco* has provided a unique set of estimates of the health burden of tobacco in all countries of the world in the year 2004 [2]. Death rates are reported for the main tobacco-related diseases and for “All causes”. In this report we find not only observed overall death rates for various causes of death but also estimates of the Population Attributable Fraction (PAF) for tobacco exposure, i.e. the part of the death rate that is specifically attributable to tobacco. Calculation of PAF can make use of data regarding prevalence of tobacco use, but such data are often absent and/or lack comparability between countries. WHO has therefore adapted a method, the PAF/SIR method (SIR: Smoking Impact Ratio), that instead of prevalence data makes use of the number of lung cancer deaths by age and sex. (Technical details are described in the report). The use of the PAF/SIR method will therefore have contributed to best possible validity of the data.

The estimated death rates attributable to tobacco do directly represent the size of each country’s health burden of tobacco with respect to different diseases and “All causes”. Estimates are given for men and women in different age groups and for the total population. The data for total population are not age standardized, so they cannot be used for fully representative comparisons between countries, but such comparisons can be made for specific age groups.

The purpose of the current study is to describe differences between Sweden and Europe as a whole with regard to mortality attributable to tobacco and to investigate how such differences are associated with national characteristics of tobacco use and tobacco control practices.

## Methods

An overview of the mortality attributable to tobacco in different EU Member States was established by extracting from the WHO Report data for each EU country regarding death rates attributable to tobacco for selected causes of death in men and women. To get comparability between countries, data for a specific age group, 60-69, were used. To illustrate the wide inter-country differences, data were presented for Sweden and for minimum, median and maximum countries outside Sweden, see Table 1.

**Table 1:** Death rates (per 100,000) attributable to tobacco

	**Sweden**	**European Union Member States other than Sweden**		
		**Min**	**Median**	**Max**
**MEN**				
Lung cancer	87	91	220	399
Other cancer	36	41	105	217
All cardiovascular	72	107	170	618
All causes	222	378	550	1388
**WOMEN**				
Lung cancer	61	5	39	127
Other cancer	17	1	10	39
All cardiovascular	63	5	50	222
All causes	173	14	115	690

Men and women age 60-69.

In order to get more detailed comparisons for some of the main causes of death we calculated, for different age groups, ratios between death rates attributable to tobacco in Sweden and corresponding rates for Europe as a whole, see Table 2. This analysis includes men only, since Swedish women, as seen in Table 1, do not differ from EU average so as Swedish men do.

**Table 2:** Ratios of death rates attributable to tobacco

**Age group**	**Cause of death**		
	**All causes**	**Lung cancer**	**All cardiovascular**
45-59	0.15	0.24	0.13
60-69	0.27	0.38	0.19
70-79	0.42	0.49	0.33

Men in Sweden vs. men in Europe as a whole 2004.

In order to account for major differences between age groups with regard to patterns of tobacco use data were retrieved from studies published by *Statistics Sweden*[3]. The data in Table 3 show how prevalence of smoking and snus use changed in different age groups up to the time represented by the WHO report. Here again the analysis includes men only.

**Table 3:** Tobacco use changes among men in Sweden

**Age group**	**Prevalence of**			
	**Daily smoking**		**Daily snus use**	
	**1988/89**	**2004/05**	**1988/89**	**2004/05**
35-44	33%	13%	19%	31%
45-54	32%	21%	11%	24%
55-64	28%	21%	9%	18%

Other potential determinants of tobacco use in different countries consist of market regulations and other kinds of tobacco control legislation. The analyses in the current study do therefore also take into account the differences between European countries with respect to tobacco control regulations. These matters are described in *The Tobacco Control Scale 2010 in Europe*, a report published by the Association of European Cancer Leagues [4].

## Findings

### Differences between countries in Europe

*The Tobacco Control Scale 2010 in Europe* presents an assessment of the implementation of selected national tobacco control policies in 30 European countries. Six policy areas were taken into account: pricing, smoking restrictions, public information, advertising bans, warning labels and treatment opportunities. The top ranking country got 77 points out of 100 possible, the lowest ranking country got 32. Sweden scored quite modestly and got just 51 points.

The WHO Report shows large differences between European Union Member States with regard to death rates specifically attributable to tobacco. For example, 60–69 year old men exhibit a variation between 72/100.000 and 618/100.000 for “All cardiovascular”. Table 1 shows, for the age group 60–69, some more comparisons between Sweden and the other European Union Member States. Among Swedish men the death rates attributable to tobacco are lower than among men in any other EU country, while Swedish women have rates similar to the European average.

### Sweden/Europe mortality comparisons by age groups

Table 2 shows, for different age groups, ratios between death rates attributable to tobacco for men in Sweden vs men in Europe as a whole. For each cause of death these ratios demonstrate substantially lower mortality attributable to tobacco for men in Sweden than for men of same age in Europe as a whole. The differences between Sweden and Europe as a whole are considerably larger in younger age groups than in older ones.

### Tobacco use changes among Swedish men in different age groups

The death rates attributable to tobacco that are presented in the WHO report are determined by the development of the tobacco use up to the year 2004. The data in Table 3 show that in all age groups the prevalence of smoking was going down, and the prevalence of snus use was going up. Thereby snus use became an increasing proportion of the total tobacco use. Further, the shift from cigarettes to snus was substantially more pronounced in younger than in older age groups.

## Discussion

The WHO Report data on mortality attributable to tobacco demonstrate that Swedish men stand out in a uniquely favourable position in comparison with other European Union Member States. What can explain this position?

The position of a country in a comparison of death rates attributable to tobacco is influenced by various national conditions with regard to political action and personal practices. Sweden’s modest scoring on *The Tobacco Control Scale 2010 in Europe* suggests that the kinds of national political action measured by that survey can not explain the uniquely low level of tobacco-related mortality for Swedish men. The main explanation must rather come from some Swedish-men-specific factor that was not taken into account in the above assessment. One such factor is the use by Swedish men of the Swedish low-toxicity oral tobacco, snus, instead of cigarettes. During the last 50 years the initially high smoking rates in men have been drastically reduced, and snus use has become the dominating kind of tobacco use among men, while female use of snus is still on a low level [5].

The age-group-specific mortality ratios Sweden vs Europe as a whole add further details regarding the relative position of Swedish men. The observation that younger age groups exhibit even more favourable positions than older ones, raises the question how differences between age groups with respect to development and levels of smoking and snus use play a role for the differences with respect to the ratios of death rates. This question can be elucidated by mortality data from the data base NORDCAN [6]. For example, data from this source show that age-specific lung cancer death rates for men in Sweden were decreasing from 1989 to 2005 by 46% in the age group 35–44, by 28% in the age group 45-54 and by 15% in the age group 55–64. This confirms the observation from Table 2 that the mortality advantage was largest in the age groups with largest dominance of snus use over smoking. This co-variation between mortality advantage and snus dominance suggests that the use of snus instead of smoking can be an important part of the explanation why younger age groups stand out even more favourably than older ones in the comparison with Europe as a whole.

## Conclusion

The low toxicity of Swedish snus is well recognized [7-9]. Consequently, there is general agreement that use of snus instead of cigarettes will be beneficial for the health of individual users. At the same time questions have been raised about the effects on population level. However, scientific studies in Sweden have found that snus use among men has contributed both to less initiation of smoking and to more cessation of smoking [10-13]. These findings in combination with the low toxicity suggest a potential for significant public health benefits [14]. The above analysis of data from the new WHO report and Swedish sources supports the assumption that the use of snus among Swedish men has yielded substantial public health benefits by contributing to make their level of mortality attributable to tobacco lowest in Europe.

## Competing interests

The authors declare that they have no competing interests. No external funding of this study. Aspects of this paper were presented in a poster at the 20th Annual International Meeting of the Society for Research in Nicotine and Tobacco (SRNT) at the Sheraton Hotel in Seattle, WA, in February 2014.

## Authors’ contributions

LR conceived the research idea and held the primary responsibility for study design. TW participated in analysis and interpretation of data and writing of the report. Both authors read and approved the final manuscript.

## Authors’ information

LR: Director, Institute for Tobacco Studies, Täby, Sweden. TW: President, Liqusa Research, Åtvidaberg, Sweden.
